# Inhibition of Oxidative Stress-Elicited AKT Activation Facilitates PPARγ Agonist-Mediated Inhibition of Stem Cell Character and Tumor Growth of Liver Cancer Cells

**DOI:** 10.1371/journal.pone.0073038

**Published:** 2013-08-30

**Authors:** Lanlan Liu, Zhaojuan Yang, Yingqian Xu, Jingyi Li, Dongxu Xu, Li Zhang, Jiabin Sun, Suhua Xia, Feiyan Zou, Yongzhong Liu

**Affiliations:** 1 State Key Laboratory of Oncogenes and Related Genes, Shanghai Cancer Institute, Renji Hospital, Shanghai Jiao Tong University School of Medicine, Shanghai, China; 2 Department of Developmental and Regenerative Biology, College of Life Science and Technology, Jinan University, Guangzhou, China; University of California, Los Angeles, United States of America

## Abstract

Emerging evidence suggests that tumor-initiating cells (TICs) are the most malignant cell subpopulation in tumors because of their resistance to chemotherapy or radiation treatment. Targeting TICs may be a key innovation for cancer treatment. In this study, we found that PPARγ agonists inhibited the cancer stem cell-like phenotype and attenuated tumor growth of human hepatocellular carcinoma (HCC) cells. Reactive oxygen species (ROS) initiated by NOX2 upregulation were partially responsible for the inhibitory effects mediated by PPARγ agonists. However, PPARγ agonist-mediated ROS production significantly activated AKT, which in turn promoted TIC survival by limiting ROS generation. Inhibition of AKT, by either pharmacological inhibitors or AKT siRNA, significantly enhanced PPARγ agonist-mediated inhibition of cell proliferation and stem cell-like properties in HCC cells. Importantly, in nude mice inoculated with HCC Huh7 cells, we demonstrated a synergistic inhibitory effect of the PPARγ agonist rosiglitazone and the AKT inhibitor triciribine on tumor growth. In conclusion, we observed a negative feedback loop between oxidative stress and AKT hyperactivation in PPARγ agonist-mediated suppressive effects on HCCs. Combinatory application of an AKT inhibitor and a PPARγ agonist may provide a new strategy for inhibition of stem cell-like properties in HCCs and treatment of liver cancer.

## Introduction

Hepatocellular carcinoma (HCC) ranks as the fifth most common cancer worldwide. However, HCC has a high rate of chemotherapy-resistance and a high incidence of recurrence and metastasis after surgical treatment, which makes it as the third leading cause of cancer mortality [1], [2]. Multiple studies have demonstrated that HCC may originate from a subpopulation of stem-like cells, called tumor-initiating cells (TICs) or cancer stem cells, which are characterized by the expression of specific surface markers and display self-renewal, differentiation, tumor initiation, and drug-resistance [3]–[10]. Although the drug sorafenib has recently been approved for HCC treatment, limited survival benefits for patients with late-stage HCC and no strong efficacy on tumor metastasis have been shown [11], [12]. Therefore, alternative therapeutic modalities to eliminate or limit the subpopulation of TICs may be an effective strategy for HCC treatment.

The peroxisome proliferator-activated receptor γ (PPARγ) is a ligand-dependent transcription factor belonging to the nuclear hormone receptor superfamily. The agonists for the activation of PPARγ include endogenous lipophilic ligands, such as 15-deoxy-Δ^12,14^-prostaglandin J_2_ (15d-PGJ_2_) and fatty acids, as well as the synthetic thiazolidinediones (a class of anti-diabetic drugs), including rosiglitazone, troglitazone, ciglitazone, pioglitazone and englitazone [13]. Ligand activation of this transcription factor leads to the expression of target genes to control many essential physiological processes, such as metabolism, cell differentiation, apoptosis, and tissue inflammation [14]. PPARγ agonists have also been shown to arrest cell proliferation, induce apoptosis, decrease cell adhesion and migration, and promote differentiation of cancer cells of different origins [15]–[22]. PPARγ blocks carcinogenesis and the invasive and metastatic potentials of HCC [23], [24], indicating that the application of PPARγ agonists may be a therapeutic prospect for HCC treatment. Interestingly, PPARγ agonists have been recently implicated in driving ET-743-mediated differentiation of myxoid round cell liposarcoma [15] and the inhibition of TICs in brain cancer [25].

In this study, we showed that PPARγ agonists (15d-PGJ_2_ or rosiglitazone) effectively inhibited stem cell-like properties in human liver cancer cells, and that NADPH oxidase-2 (NOX2)-induced reactive oxygen species (ROS) generation functioned as a key downstream event. As a negative feedback response, increased ROS elicited hyperactivation of AKT, which significantly counteracted PPARγ agonist-mediated inhibition of stem cell-like properties in HCC cells. *In vivo* experiments further showed that the disruption of the negative feedback loop by the AKT inhibitor triciribine significantly facilitated the PPARγ agonist rosiglitazone-mediated inhibition of tumor growth. These findings suggest that the combination of an AKT inhibitor and a PPARγ agonist may provide a promising potential treatment for liver cancer.

## Materials and Methods

### Ethics Statement

All animal experimental protocols were approved by the Medical Experimental Animal Care Committee of Shanghai Cancer Institute (Approval ID. ShCI-11-020).

### Cell Culture

SK-Hep1 and Hep3B cell lines were obtained from American Type Culture Collection (ATCC, Manassas, VA). Huh7 cell line was from Riken Cell Bank (Tsukuba Science City, Japan). SMMC 7721 cell line was provided by the Department of Pathology of the Second Military Medical University (Shanghai, China) [26]. All cell lines were cultured in DMEM with high glucose (GIBCO, Grand Island, NY) supplemented with 10% fetal bovine serum (GIBCO) and penicillin/streptomycin (1% [v/v]; GIBCO) at 37°C in a humidified 5% CO_2_ atmosphere. After cells were initially grown, multiple aliquots were cryopreserved and all cell lines were used within 6 months after resuscitation.

For treatment experiments, cells were plated and grown over night, the medium was then replaced with high-glucose DMEM medium containing 1% fetal bovine serum, and incubated with 15d-PGJ_2_ (Sigma-Aldrich, St. Louis, MO), rosiglitazone (Cayman Chemical, Ann Arbor, MI), N-acetylatedcysteine (NAC) (Calbiochem, Darmstadt, Germany), triciribine (Santa Cruz Biotechnology, Santa Cruz, CA), and/or LY294002 (Sigma-Aldrich), for the indicated times. All experiments were conducted three times.

### Fluorescence-activated Cell Sorting (FACS) Analysis

After incubation under indicated culture conditions, cells were dissociated and washed twice with PBS containing 0.5% BSA at 4°C. PE-conjugated anti-human CD133 antibody (Miltenyi Biotec, Bergisch Gladbach, Germany) was added for incubation at 4°C for 30 minutes. Flow cytometry was performed on FACSCalibur flow cytometer (BD Biosciences, San Jose, CA). Rat IgG1/κ antibody conjugated to phycoerythrin served as an isotype control. Dead cells was excluded by staining with 7-AAD (Sigma-Aldrich) before analysis. For cell sorting, CD133^+^ or GFP^+^ cells were stringently gated and isolated using a MoFlo XDP (Beckman Coulter, Fullerton, CA).

### Cell Viability Assay

Cell viability was determined by 3-(4,5-dimethyl-2-thiazolyl)-2,5- diphenyl-2H-tetrazolium bromide (MTT) (Sigma-Aldrich) method. In brief, a total of 1000 cells/well were seeded into 96-well plate in a final volume of 200 µl. After incubation with 15d-PGJ_2_ for the indicated times, 20 µl MTT solution (5 mg/ml in PBS) was added to the medium and cultured for additional 3 hours. Then, the MTT solution was discarded and 150 µl dimethyl sulfoxide (DMSO, Sigma-Aldrich) was added into each well. The absorbency of each well was measured at a wavelength of 540 nm.

### Apoptosis Assay

The extent of apoptosis was evaluated by Pharmingen™ FITC Annexin V Apoptosis Detection Kit (BD Biosciences) according to the provided manufacturer's instructions. Then, Fluorescence-activated cell sorting analysis was conducted on the FACSCalibur flow cytometer (BD Biosciences). Single staining using Annexin V-FITC or 7-AAD alone was performed as controls.

### BrdU Assay

Pharmingen™ APC BrdU Flow Kit (BD Biosciences) was used for Bromodeoxyuridine (BrdU) incorporation assay according to the manufacturer’s instructions.

### RNA Extraction and Real-time PCR

Total RNA was isolated from cells with RNAiso Reagent (TaKaRa, Dalian, China). Reverse transcription (RT) was carried out using 500 ng of total RNA for cDNA synthesis in a 10 µl reaction volume, using the PrimeScript™ RT reagent kit (TaKaRa) according to the manufacturer’s instructions. Using Premix Ex Taq™ (TaKaRa), quantitative PCR was performed for *Nanog*, *OCT4*, and *AFP*. For detection of *Notch1*, *SMO*, *NOX2, NOX4, P22^phox^, P47^phox^, P67^phox^* and *Rac* expression, quantitative real-time PCR was carried out using SYBR green mix from TaKaRa on a 7300 Real-Time PCR System (Applied Biosystems, Foster City, CA). *GAPDH* was used as an internal control. The primers are listed in Table S1.

### Small Interfering RNA (siRNA) Transfection

The siRNAs specific to *NOX2*, *PPARγ*, *AKT1* and *AKT2* were purchased from Sigma-Aldrich. The siRNA sequences are listed in Table S2. One day before the transfection, cells were seeded into six-well plates without antibiotics. The siRNAs (60 nM) were transfected into cells with Lipofectamine 2000 (Invitrogen, Carisbad, CA) according to the manufacturer's instructions.

### Western Blotting Analysis

Cells were lysed in a RIPA lysis buffer (Beyotime, Nantong, China) containing Protease Inhibitor Cocktail and PhosSTOP Phosphatase Inhibitor (Roche, Monza, IT). Proteins were analyzed using indicated antibodies: anti-CD133, anti-PPARγ, anti-AKT, anti-phospho-AKT (Ser473) (all from Cell Signaling Technology, Beverly, MA); anti-GAPDH, anti-α-tubulin (all from Santa Cruz Biotechnology, Santa Cruz, CA); and anti-NOX2 (Abcam, Cambridge, UK). The ChemiDoc™ XRS system (Bio-Rad Laboratories, Hercules, CA) was used to obtain images.

### Spheroid-forming Assay

Single cells were seeded into ultra-low attachment culture dishes (Costar, Coring, NY) at a density of 500 cells/ml in a serum-free 1∶1 DMEM:F12 (GIBCO), supplemented with B27 (1∶50; GIBCO), 20 ng/ml epidermal growth factor (EGF) and 20 ng/ml basic fibroblast growth factor (bFGF) (R&D Systems, Minneapolis, MN). All cultures were incubated for 7 days and spheroid counts were performed.

### Measurement of ROS Accumulation

The oxidation-sensitive fluorescent probe Dihydroethidium (DHE) (Invitrogen) was used to analyze the intracellular level of ROS. Cells were washed with PBS and incubated with 5 µM DHE for 30 minutes in a 37°C incubator. Fluorescence was measured by a FACSCalibur flow cytometer.

### *In vivo* Experiments

Male nude BALB/c mice, 6-week-old, were housed under pathogen-free conditions, and all experiments involving mice were performed according to the Institutional Animal Care and Use Committee of Shanghai and the National Research Council Guide for Care and Use of Laboratory Animals.

Huh7 cells with GFP expression (2×10^6^) were suspended in 100 µl PBS and inoculated subcutaneously (s.c.) into the right flank of male nude mice. The subcutaneous models were administered drugs beginning at day 4 following tumor cell injection. Rosiglitazone (GlaxoSmithKline, Marly le Roi, France) was mixed with sterile water, at a concentration of 4 mg/ml. Triciribine (Santa Cruz Biotechnology) was dissolved in PBS, at a concentration of 200 µg/ml. For single treatment, the subcutaneous mice were randomized to vehicle and rosiglitazone treatment group (n = 7 per group). Rosiglitazone (100 mg/kg) was administered daily intragastrically (ig) for 12 days. For combination treatment, the subcutaneous mice were randomly assigned to 4 experimental groups (n = 5 per group). The 4 experimental groups were as follows: untreated control group, rosiglitazone treatment group (100 mg/kg/day, administered at day 6, 7, 10, 11 by ig), triciribine treatment group [1 mg/kg/day, administered at day 4, 5, 8, 9, 12, 13 by intraperitoneally (i.p.)], and rosiglitazone-combined triciribine treatment group (administered by using the same schedule for each drug as described for the single treatment). All control mice were administered with an equal volume of vehicle by gavage or injection, respectively. Tumor size, measured individually daily with microcalipers after treatment, was calculated according to the formula: *V = *(*w*_1_×*w*_2_×*w*_2_)/2, in which *w*_1_ is the length and *w*_2_ is the width of tumor. All mice were killed by cervical dislocation the day after the last treatment. Tumor masses were surgically excised, weighed grossly, and dissociated into single cells as previously described [27]. Cells were then stained with PE-conjugated anti-human CD133 antibody (Miltenyi Biotec) for determining CD133 expression in GFP^+^ tumor cells, or GFP^+^ tumor cells isolated by flow sorting were used to assess spheroid-forming ability.

### Statistical Analysis

Statistical analysis was carried out with the Graphpad Prism software. Statistical differences between two groups were determined using the student's *t* test. The criterion for statistical significance was *P* value less than 0.05.

## Results

### PPARγ Agonists Inhibit the Stem Cell-like Properties and Tumor Growth of HCC Cells

**Figure 1:**
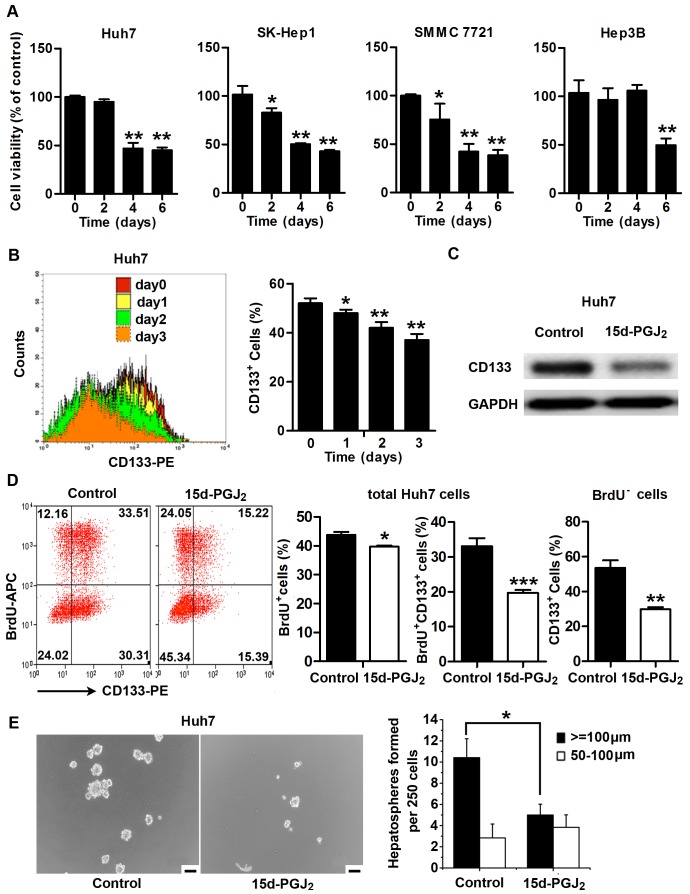
PPARγ agonist 15d-PGJ_2_ inhibits the stem cell-like properties of HCC cells *in vitro*. **A**, Huh7, SK-Hep1, SMMC 7721 and Hep3B cells were treated with 0.5 µg/ml 15d-PGJ_2_ for the indicated times. Cell proliferation was evaluated by MTT assay. *, *P*<0.05; **, *P*<0.01, versus control cells. **B**, Huh7 cells were treated with 0.5 µg/ml 15d-PGJ_2_ for the indicated times and stained with CD133-PE. Before analysis, 7-AAD was used to exclude dead cells. Representative FACS profiles are shown (left panel). Percentages of CD133^+^ cells are shown as the means ± S.E.M (n = 3) (right panel). *, *P*<0.05; **, *P*<0.01. **C**, Huh7 cells were treated with 0.5 µg/ml 15d-PGJ_2_ for 24 hours and total protein was extracted for analysis of CD133 expression. All samples were normalized to GAPDH expression. **D**, Huh7 cells were treated with 0.5 µg/ml 15d-PGJ_2_ for 24 hours. Cells were harvested for BrdU incorporation and CD133 staining. Quantitative bar graphs are presented as the means ± S.E.M (n = 3). *, *P*<0.05; **, *P*<0.01; ***, *P*<0.001, versus control cells. **E**, Huh7 cells were cultured in spheroid-forming medium containing 15d-PGJ_2_ (1 µg/ml) for 7 days. Representative microscopic pictures are shown (left panel). Scale bar = 100 µm. Quantitative bar graphs are shown as the means ± S.E.M. (n = 3) (right panel). *, *P*<0.05.

We first tested whether PPARγ agonists inhibited cell proliferation in the HCC cells. Consistent with previous observations that PPARγ agonists usually inhibit the proliferation of tumor cells, we found that treatment with 15d-PGJ_2_, a natural endogenous ligand of PPARγ, significantly inhibited cell proliferation in all examined HCC cell lines (Figure 1A). We further examined the expression of stemness-related genes in four HCC cell lines, Huh7, SK-Hep1, SMMC 7721, and Hep3B cells, with or without 15d-PGJ_2_ treatment. As shown in Figure S1A, a number of stemness-related genes, including *Nanog*, *Notch1*, *OCT4*, and *SMO*, showed a tendency toward downregulation in all 15d-PGJ_2_-treated cells, indicating that PPARγ agonists may have an effect on HCC TICs. Previous studies have demonstrated that the CD133^+^ subpopulation in Huh7 cells represents a pool of cells containing TICs and is essential for driving tumor growth [28], [29]. We therefore analyzed CD133 expression, spheroid-forming ability, and *in vivo* tumorigenicity of Huh7 cells after treatment with PPARγ agonists. We found a slight increase in apoptosis of Huh7 cells upon treatment with 15d-PGJ_2_ for 48 hours (Figure S1B). Interestingly, after using 7-AAD staining to exclude dead cells, we observed a pronounced decrease in the percentage of CD133^+^ cells in 15d-PGJ_2_-treated cells (Figure 1B). The decreased expression of CD133 protein in 15d-PGJ_2_-treated Huh7 cells was further confirmed by Western blotting analysis (Figure 1C). We also examined the PPARγ agonist-treated cells for BrdU incorporation and CD133 staining, and we found that the percentage of BrdU-labeled CD133^+^ cells was significantly decreased after treatment with 15d-PGJ_2_. Moreover, in the subpopulation negative for BrdU staining, a dramatic decrease in CD133 expression was also observed (Figure 1D). In addition, treatment with 1 µg/ml 15d-PGJ_2_ significantly reduced spheroid numbers of Huh7 cells (Figure 1E).

**Figure 2:**
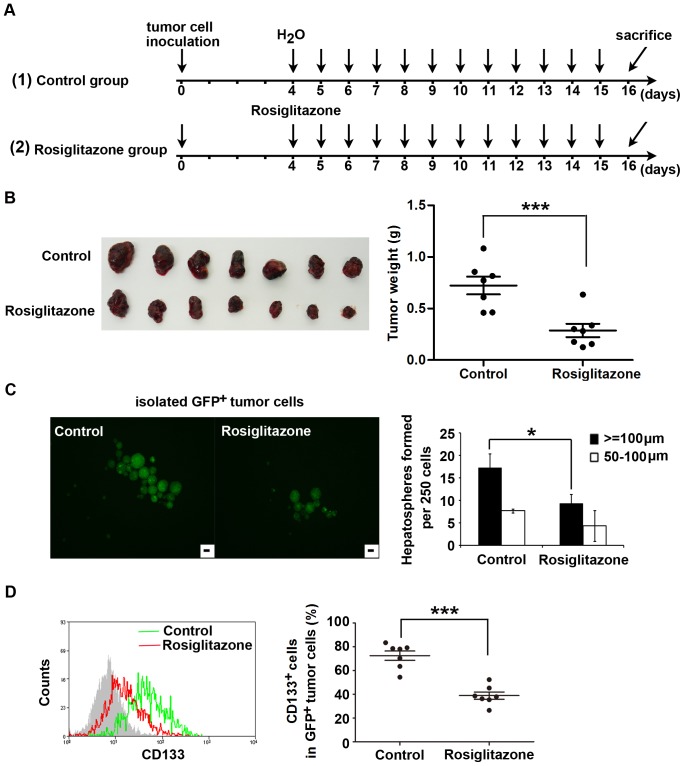
Rosiglitazone treatment reduces pool size of the CD133^+^ cell fraction and inhibits tumor growth of Huh7 cells *in vivo*. GFP^+^ Huh7 cells (2×10^6^) were s.c. inoculated into nude mice. After 4 days, mice with tumors were treated daily with vehicle or rosiglitazone (100 mg/kg/day) by ig for 12 days (n = 7 per group). **A**, Schematic outline of the experimental setup. **B**, Original images of xenograft tumors are shown (left panel). Tumor weight was measured at the end of treatment (right panel). Each dot represents the weight of an individual xenograft tumor. Horizontal bars represent the mean ± SD (n = 7). ***, *P*<0.001. **C**, Tumor tissues from control and treated groups were enzymatically dissociated into single-cell suspensions and GFP^+^ tumor cells were harvested by flow sorting and cultured in spheroid-forming medium for 7 days. A representative photograph is shown (left panel). Scale bar = 100 µm. A summary bar graph (mean ± SD) is shown in the right panel. *, *P*<0.05. **D**, The dissociated single tumor cells from each mouse were stained with CD133-PE. The percentage of GFP^+^CD133^+^ cells was determined by flow cytometry. Representative flow cytometry analysis profiles are shown (left panel). The proportion of CD133^+^ cells in GFP^+^ tumor cells is presented as the mean ± SD (right panel). ***, *P*<0.001.

To substantiate our observations with *in vivo* experiments, we treated mice bearing GFP^+^ Huh7 tumors with a PPARγ agonist. Because the biological activity of 15d-PGJ_2_ is usually limited *in vivo* [30], [31], rosiglitazone, a synthetic PPARγ agonist, was used in *in vivo* experiments at a dose of 100 mg/kg/day (the treatment schedule shown in Figure 2A). An antitumor growth effect of rosiglitazone was observed (Figure 2B). Tumor growth was inhibited by approximately 60% (*P*<0.001). We further assessed the spheroid-forming potential of tumor cells derived from control and rosiglitazone-treated mice. Remarkably, GFP^+^ tumor cells isolated from primary tumors in rosiglitazone-treated mice formed smaller and fewer spheres than those from the control group (Figure 2C). Administration of 100 mg/kg/day rosiglitazone also significantly decreased the proportion of GFP^+^CD133^+^ tumor cells (Figure 2D). These results suggest a prominent inhibitory effect of PPARγ agonist on tumor growth and stem cell-like properties of HCC Huh7 cells *in vivo*.

### PPARγ Agonists Inhibit Cancer Stem Cell-like Phenotypes via NOX2-dependent ROS Generation

**Figure 3:**
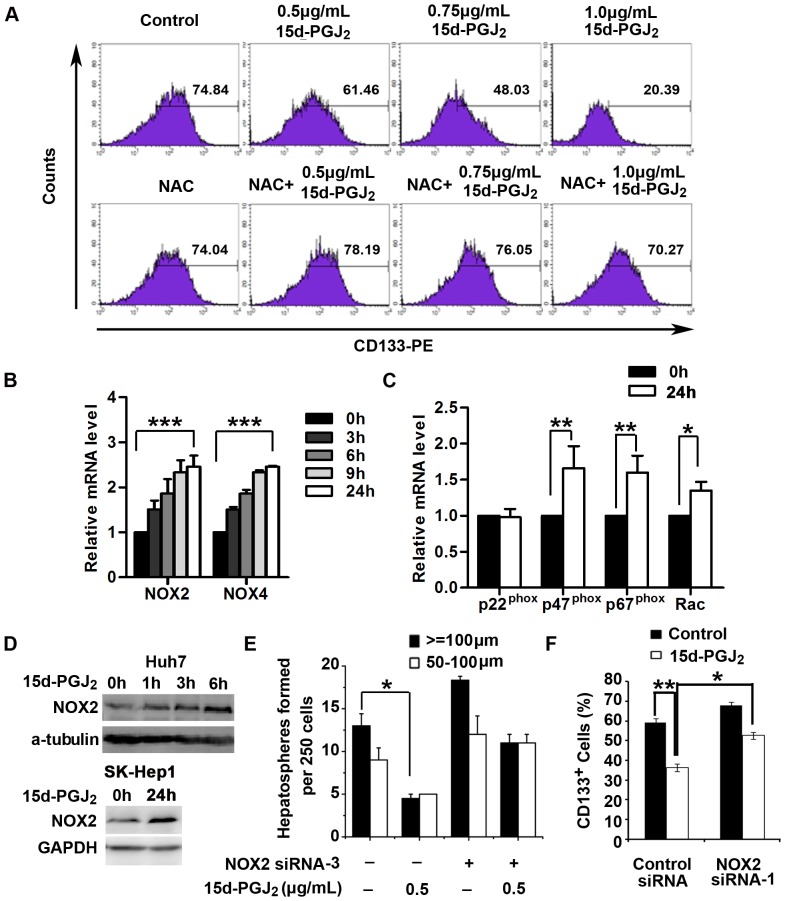
PPARγ agonist inhibits cancer stem cell-like properties through NOX2-mediated ROS generation. **A**, Isolated CD133^+^ Huh7 cells were treated with 15d-PGJ_2_ (0.5, 0.75 or 1.0 µg/ml) and NAC (10 mM) either alone or in combination, and the percentage of CD133^+^ cells was detected by flow cytometry after 72 hours. **B**, Huh7 cells were treated with 0.5 µg/ml 15d-PGJ_2_ for the indicated times. The expression of mRNA of NADPH oxidase genes (*NOX2* and *NOX4*) was measured by quantitative RT-PCR. Data are means ± S.E.M. (n = 3). ***, *P*<0.001. **C**, Huh7 cells were treated with 0.5 µg/ml 15d-PGJ_2_ for 24 hours. The expression *P22^phox^, P47^phox^, P67^phox^* and *Rac* mRNA was determined by quantitative RT-PCR. *, *P*<0.05; **, *P*<0.01. **D**, Huh7 and SK-Hep1 cells were treated with 0.5 µg/ml 15d-PGJ_2_ for the indicated times and total protein was extracted for analysis of NOX2 expression. **E**, Huh7 cells were transiently transfected with negative control siRNA or NOX2 siRNA-3, after which cells were seeded on ultra-low attachment culture dishes and cultured in spheroid-forming medium containing 15d-PGJ_2_ for 7 days. Number of spheroids formed is shown as the means ± S.E.M. (n = 3). *, *P*<0.05. **F**, Huh7 cells were transiently transfected with negative control siRNA or NOX2 siRNA-1. Cells were treated with 0.5 µg/ml 15d-PGJ_2_ for 72 hours and stained with CD133-PE. Data are means ± S.E.M. (n = 3). *, *P*<0.05; **, *P*<0.01.

It has been reported that PPARγ agonists induce ROS production in multiple types of cancer cells [32], [33]. Since the regulation of cell differentiation has been observed to be a critical function of ROS [34], [35], we investigated the possibility that ROS production contributed to PPARγ agonist-induced repression of stem cell-like properties in HCC cells. We first tested ROS levels in HCC cells after exposure to 15d-PGJ_2_. The results showed that the levels of intracellular ROS were dramatically increased in both Huh7 and SK-Hep1 cells after treatment with 15d-PGJ_2_ for 72 hours (Figure S2A). We further isolated CD133^+^ subpopulations from Huh7 cells by FACS sorting and cultured them in the presence or absence of 15d-PGJ_2_ and the antioxidant NAC, a precursor of intracellular glutathione that inhibits ROS production. After 3 days in culture, the percentage of CD133^+^ cells in the control cells was reduced to 74.84%, whereas 15d-PGJ_2_ treatment caused significantly larger decreases in the proportion of CD133^+^ cells in a dose-dependent manner (Figure 3A). Strikingly, the frequency of CD133^+^ cells was decreased to 20.39% when the concentration of 15d-PGJ_2_ was 1.0 µg/ml. However, NAC treatment robustly attenuated this 15d-PGJ_2_-mediated inhibition and preserved the CD133^+^ compartment (Figure 3A). Consistent with this observation, the inhibitory effect of 15d-PGJ_2_ on spheroid formation was dramatically diminished when Huh7 cells were pretreated with NAC (Figure S2B). Moreover, NAC also attenuated the decrease in expression of stemness-related genes in both Huh7 and SK-Hep1 cells (Figure S2C). These observations suggest that PPARγ agonists inhibited the cancer stem cell-like phenotypes through positive regulation of ROS production. In addition, depletion of PPARγ by siRNA had only subtle effects on the ROS generation induced by 15d-PGJ_2_ (Figure S3), indicating that PPARγ treatment results in ROS induction mostly through a PPARγ-independent pathway.

The NADPH oxidase (NOX) family of proteins is responsible for ROS production [36]. To determine whether NADPH oxidases are upregulated and contributable to the increased ROS generation by the stimulation of PPARγ agonists, we analyzed the abundance of various *NOX* mRNAs, including *NOX2* and *NOX4*, in cells treated with 15d-PGJ_2_. The expression of *NOX2* and *NOX4* were promptly upregulated, starting approximately 3 hours after treatment with 15d-PGJ_2_ (Figure 3B). Furthermore, we found that transfection of siRNAs specific for *NOX4* did not impair 15d-PGJ_2_-induced ROS generation (data not shown), whereas the mRNA levels of several major NOX2 components, such as *P22*, *P47*, *P67* and *Rac*, were significantly increased in 15d-PGJ_2_-treated Huh7 cells (Figure 3C). Additionally, the protein level of NOX2 was notably elevated in both Huh7 and SK-Hep1 cells upon treatment with 15d-PGJ_2_ (Figure 3D). We further depleted NOX2 expression with specific siRNAs in both Huh7 and SK-Hep1 cells, and found that NOX2-depleted cells exhibited a weaker induction of ROS in response to 15d-PGJ_2_ treatment compared with cells transfected with control siRNA (Figure S4A). The efficiency of *NOX2* siRNAs at inhibiting NOX2 expression was validated by Western blotting and quantitative RT-PCR analysis (Figure S4B and C). Indeed, downregulation of NOX2 significantly counteracted 15d-PGJ_2_-mediated inhibition of spheroid formation and pool size of the CD133^+^ subset in Huh7 cells (Figure 3E and F). Together, these results indicate that upregulation of NOX2 was responsible for PPARγ agonist-mediated inhibition of stem cell-like properties in HCC cells.

### Reciprocal Regulation of ROS Generation and AKT Activation in PPARγ Agonist-treated HCC Cells

**Figure 4:**
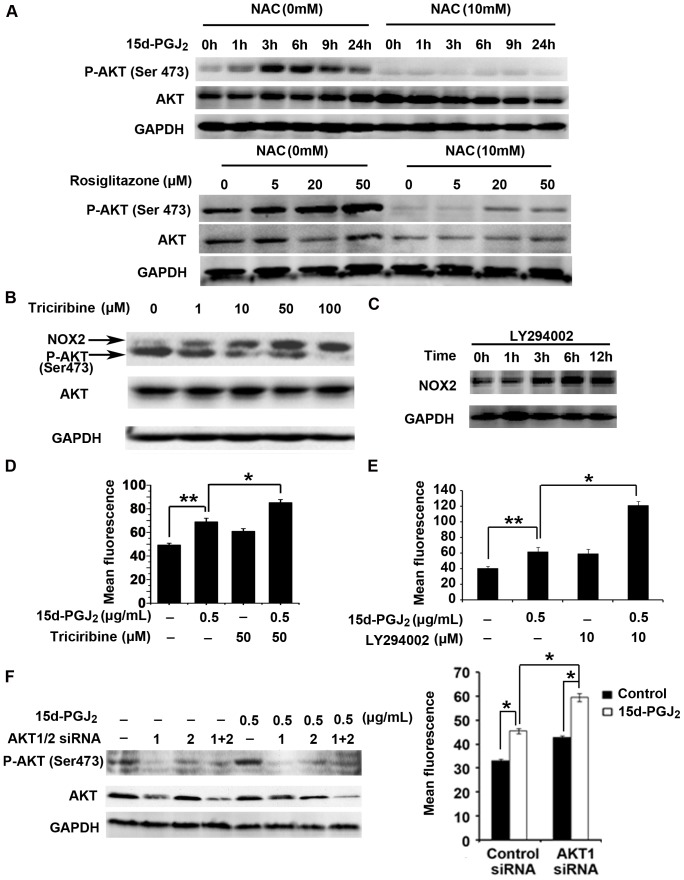
Reciprocal regulation of ROS generation and AKT activation in PPARγ agonist-treated Huh7 cells. **A**, Huh7 cells were pretreated with NAC (10 mM) for 30 min followed by 15d-PGJ_2_ (0.5 µg/ml) for the additional indicated times (top panel). Huh7 cells were treated with rosiglitazone (5, 20 or 50 µM) and NAC (10 mM) either alone or in combination for 6 hours (bottom panel). Total protein was extracted for analysis of P-AKT, AKT, and GAPDH expression. **B**, Huh7 cells were treated with the AKT inhibitor triciribine for 3 hours and total protein was extracted for analysis of NOX2, P-AKT, AKT, and GAPDH expression. **C**, Huh7 cells were treated with LY294002 (10 µM) for the indicated times, and the protein level of NOX2 was examined by Western blotting analysis. **D**, Huh7 cells were treated with 15d-PGJ_2_ and triciribine either alone or in combination for 48 hours, after which they were labeled with DHE and analyzed by flow cytometry. Data are means ± S.E.M. (n = 3). *, *P*<0.05; **, *P*<0.01. **E**, Huh7 cells were treated with 15d-PGJ_2_ and LY294002 either alone or in combination for 72 hours, after which they were labeled with DHE and analyzed by flow cytometry. Data are means ± S.E.M. (n = 3). *, *P*<0.05; **, *P*<0.01. **F**, Huh7 cells were transiently transfected with AKT1 siRNA and AKT2 siRNA either alone or in combination, after which the cells were treated with 0.5 µg/ml 15d-PGJ_2_ for another 3 hours (left panel). The levels of P-AKT and AKT proteins were measured by Western blotting analysis. Huh7 cells were transiently transfected with negative control siRNA or AKT1 siRNA, after which cells were treated with 0.5 µg/ml 15d-PGJ_2_ for another 72 hours (right panel). Intracellular ROS production was analyzed by flow cytometry (means ± S.E.M, n = 3). *, *P*<0.05.

Next, we explored possible alterations in the signaling pathways that regulate the effects mediated by the PPARγ agonists. Intriguingly, we found that both 15d-PGJ_2_ and rosiglitazone dramatically enhanced the phosphorylation of AKT, and this PPARγ agonist-induced activation of AKT was attenuated by NAC in Huh7 cells (Figure 4A). A similar phenomenon also occurred in SK-Hep1 cells (data not shown). These data indicate that PPARγ agonist-induced ROS caused AKT hyperactivation. To examine the potential relationship between AKT activation and ROS production, we used the AKT inhibitor triciribine and the PI3K inhibitor LY294002 to repress AKT activity, and we found that a dose- or time-dependent repression of AKT phosphorylation was paralleled by a gradual incremental change in NOX2 expression (Figure 4B and C). Moreover, combinatorial treatment of Huh7 cells with both 15d-PGJ_2_ and triciribine led to a higher level of intracellular ROS than either treatment alone (Figure 4D). This synergistic effect was also observed in cells co-treated with 15d-PGJ_2_ and LY294002 (Figure 4E). We further confirmed our observations using specific siRNAs against AKT1 and AKT2. The siRNA against AKT1 further increased ROS induction in the presence of 15d-PGJ_2_ (Figure 4F). The efficiency of AKT1 and AKT2 siRNAs at inhibiting total and phosphorylated AKT expression was validated by Western blotting analysis (Figure 4F). Taken together, these results suggest that PPARγ agonist-induced AKT activation modulated NOX2-mediated ROS generation through negative feedback.

### The AKT Inhibitor Triciribine Cooperates with PPARγ Agonists to Inhibit Cancer Stem Cell-like Phenotypes and Tumor Growth

**Figure 5:**
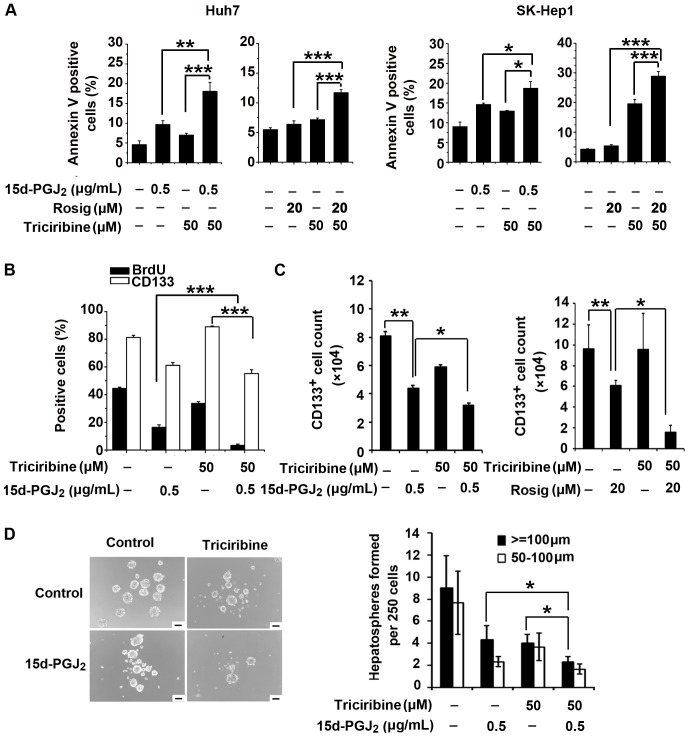
AKT inhibition facilitates the PPARγ agonist-mediated inhibitory effects on HCC cells *in vitro*. **A**, Huh7 and SK-Hep1 cells were treated with 15d-PGJ_2_, rosiglitazone and triciribine either alone or in combination. The percentage of apoptotic cells was evaluated 48 hours later by Annexin V-FITC/7-AAD staining. Data are means ± S.E.M. (n = 3). *, *P*<0.05; **, *P*<0.01; ***, *P*<0.001. **B**, Huh7 cells were treated with 15d-PGJ_2_ and triciribine either alone or in combination. The percentages of CD133^+^ cells and BrdU positive cells were analyzed 48 hours later. Quantitative data are the means ± S.E.M. (n = 3). ***, *P*<0.001. **C**, Huh7 cells were treated with 15d-PGJ_2_ and triciribine either alone or in combination (left panel). Huh7 cells were treated with rosiglitazone and triciribine either alone or in combination (right panel). The number of CD133^+^ cells was evaluated 72 hours later. Data are the means ± S.E.M. (n = 3). *, *P*<0.05; **, *P*<0.01. **D**, Huh7 cells were cultured in spheroid-forming medium containing 15d-PGJ_2_ and triciribine either alone or in combination for 7 days, after which spheroid formation was examined. Representative microscopic pictures are shown (left panel). Scale bar = 100 µm. Quantitative bar graphs (means ± S.E.M., n = 3) are shown (right panel). *, *P*<0.05.

The aforementioned results prompted us to speculate that the combined treatment of a PPARγ agonist and an AKT inhibitor may inhibit tumor growth more efficiently than either reagent alone. We first evaluated whether triciribine increased the sensitivity of HCC cells to PPARγ agonist-induced apoptosis. Indeed, in both Huh7 and SK-Hep1 cells, the combination of triciribine with 15d-PGJ_2_ or rosiglitazone markedly enhanced the induction of apoptosis compared with either alone (Figure 5A). Interestingly, we found that combination treatment of Huh7 cells with both 15d-PGJ_2_ and triciribine did not cause a further decrease in the proportion of CD133^+^ cells compared with treatment of 15d-PGJ_2_ alone. However, the combination treatment was more effective at inhibiting cell proliferation in Huh7 cells compared with treatment of either individual agent alone (Figure 5B), indicating that the combination treatment further decreased the absolute number of CD133^+^ Huh7 cells. In the cells treated with both 15d-PGJ_2_ and triciribine, we found that the number of CD133^+^ cells was reduced by 70.9%, which was higher than the 46.1% in 15d-PGJ_2_-treated cells (Figure 5C). Similarly, the number of CD133^+^ cells was reduced by 89.6% with combination treatment of rosiglitazone and triciribine, higher than the 37.5% with rosiglitazone treatment alone (Figure 5C). In addition, combination treatment suppressed spheroid formation of Huh7 cells more substantially than either 15d-PGJ_2_ or AKT inhibitor alone (Figure 5D). These data indicate that AKT inhibition by triciribine may have exacerbated the inhibitory effects of PPARγ agonists on the population of CD133^+^ cells by directly inducing cell death.

**Figure 6:**
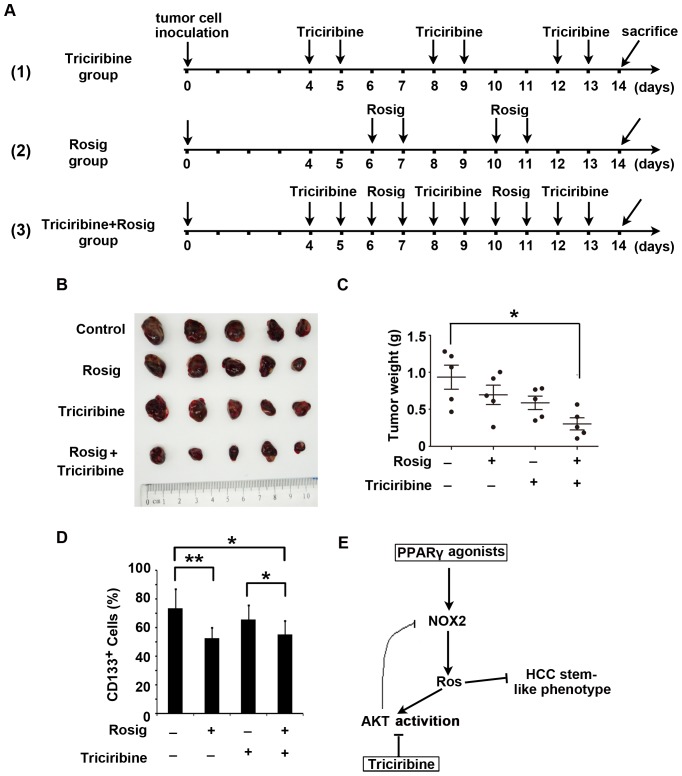
The combined treatment of rosiglitazone and triciribine synergistically represses tumor growth *in vivo*. GFP-expressing Huh7 cells (2×10^6^) were subcutaneously injected into nude BALB/c mice (n = 5 per group). After 4 days of tumor implantation, the animals were treated with rosiglitazone (Rosig, 100 mg/kg/day, ig), triciribine (1 mg/kg/day, i.p.), or the sequential combination of the drugs and then euthanized 10 days later. **A**, Schematic outline of the experimental setup. **B**, Original images of tumors from control and treated groups. **C**, Tumor weight was measured at the end of treatment. Each dot represents weight of an individual xenograft tumor. Horizontal bars represent the mean ± SD. *, *P*<0.05. **D**, Tumor tissues from control and treated groups were enzymatically dissociated into single-cell suspensions and stained with CD133-PE. The percentage of GFP^+^CD133^+^ cells was determined by flow cytometry. Data are mean ± SD. *, *P*<0.05; **, *P*<0.01. **E**, Schematic illustration of a negative feedback loop between oxidative stress and AKT hyperactivation in PPARγ agonist-treated cells.

Next, we evaluated the anti-tumor activity of the combination therapy of rosiglitazone and triciribine in a mouse xenograft tumor model. The mice inoculated with GFP^+^ Huh7 cells (2×10^6^) were assigned to one of 4 groups: no treatment, rosiglitazone (100 mg/kg/day, ig), triciribine (1 mg/kg/day, i.p.), or treatment with both rosiglitazone and triciribine sequentially. To allow for concurrent usage without exacerbating toxicity, rosiglitazone and triciribine were administered intermittently. The treatment schedule is shown in Figure 6A: rosiglitazone was given on days 6, 7, 10 and 11 and triciribine, on days 4, 5, 8, 9, 12 and 13. The total amount of rosiglitazone used in this setting was reduced by approximately 67% for each mouse compared with that used in the experiments described in Figure 2. Indeed, during the course or at the end of treatment, there was no significant difference in body weight among the four experimental groups (data not shown), indicating that there were no obvious toxic effects of the combination therapy on the mice. After 10 days of treatment, the growth of Huh7 tumors was dramatically repressed in the group with combinatorial therapy (Figure 6B). The weight of the subcutaneous xenografts in the mice with sequential combination treatment was significantly decreased compared with those in the other 3 groups (Figure 6C). In addition, the proportion of GFP^+^CD133^+^ tumor cells in the xenotransplants was examined via FACS analysis. Notably, although treatment with rosiglitazone alone did not decrease tumor size, possibly owing to the short period of treatment, the proportion of CD133^+^ cells in GFP^+^ tumor cells still significantly declined. Importantly, we did observe a decreased percentage of CD133^+^ cells in tumor tissues of the group receiving combination therapy (Figure 6D). Collectively, combination treatment of rosiglitazone and triciribine synergistically restrained the pool size of CD133^+^ tumor cells and inhibited tumor growth of HCC Huh7 cells.

## Discussion

Tumor-initiating cells are a subset of cancer cells that exhibit stem cell-like features, such as self-renewal and the capability to differentiate [37]. Evidence for the existence of TICs has been increasingly identified in many cancer types, including breast, brain, skin, prostate, colon and liver cancers [37]–[39]. Using lineage tracing techniques, researchers have recently provided the first hard evidence that TICs exist and can be induced *de novo* during tumor formation; more importantly, such cells have been shown to be a legitimate therapeutic target [40]–[42].

In the present study, we demonstrated that PPARγ agonists promoted the differentiation of CD133^+^ cells into CD133^−^ cells and inhibited the stem cell-like properties of HCC cells. This effect of PPARγ agonists on HCC TICs was dependent on NOX2-mediated ROS generation. Our results find the support from the previous study showing that TICs or cancer stem cells usually have lower levels of ROS compared with corresponding non-tumorigenic cells [43]. Because the effects of ROS are determined by the balance between production and detoxification of ROS in cells, it is still unclear whether PPARγ agonists could regulate the levels of detoxifying enzymes, such as superoxide dismutase, catalase, and glutathione peroxidase, in HCC cells. Nevertheless, our present study clearly indicates that oxidative stress induced by PPARγ agonists is mainly responsible for the inhibition of the subpopulation of TICs in HCC cells.

Moreover, our observations indicate that PPARγ agonist-induced ROS generation enhanced AKT phosphorylation, which significantly impaired the inhibitory effect of PPARγ agonists on cell proliferation and the stem cell-like phenotype of HCC cells. More importantly, AKT hyperactivation, as a feedback regulator, negatively modulated intracellular ROS production induced by PPARγ agonists. Previous study has shown that increased levels of phosphorylated AKT (Ser437) may contribute to reductions of putative death-promoting δPKC activity [44]. PKC, as key regulator of NADPH oxidases, has an important function in ROS generation. Inhibition of PKC leads to decreased expression of NOX2 and NOX4 mRNAs [45]. Thus, whether PKC participates in AKT-inhibited regulation of NOX2 expression in HCC cells requires further investigation. Furthermore, the activation of AKT that functions as a compensatory prosurvival mechanism in rat hepatocytes in response to oxidative stimuli, can suppress ROS production by inhibiting function of pro-apoptotic, prooxidative Rac1 GTPase [46], which is necessary for activation of NOX2. Interestingly, another study suggested that 15d-PGJ_2_ induces apoptosis through ROS-mediated inactivation of AKT in leukemia and colorectal cancer cells [33]. The different function of ROS on activation of AKT may due to the application in different type cells. In this paper, our data were validated by the observation that cotreatment of PPARγ agonists and the AKT inhibitor triciribine strongly sensitized HCC cells to apoptosis and inhibited their cancer stem cell-like phenotype. These results are consistent with the report that the preferential expression of AKT/PKB renders CD133^+^ HCC cells resistant to chemotherapy [47].

Notably, the AKT inhibitor triciribine alone had a cytostatic effect on tumor cells but did not preferentially inhibit CD133^+^ tumor-initiating cells, whereas the PPARγ agonist rosiglitazone selectively targeted HCC TICs. More importantly, disruption of the feedback counterbalance mediated by AKT hyperactivation further potentiated ROS-mediated damage induced by PPARγ agonists in HCC cells. Therefore, we propose that combination treatment of a PPARγ agonist and triciribine may constitute a more effective strategy for HCC therapy. Indeed, conclusive results obtained from the *in vivo* experiments demonstrated that combination treatment of rosiglitazone and triciribine dramatically impeded the growth of HCC compared with either treatment alone.

In conclusion, our study showed that PPARγ agonists repressed the stem cell-like phenotype of HCC via NOX2-mediated oxidative stress; this effect, however, was partially attenuated by the activation of AKT, indicating a negative feedback loop between oxidative stress and AKT hyperactivation during a PPARγ agonist-mediated suppressive event (Figure 6E). Combination treatment with the PPARγ agonist rosiglitazone and the AKT inhibitor triciribine synergistically inhibited the HCC stem cell-like phenotype and tumor growth *in vivo.* These findings raise the possibility that the combined administration of rosiglitazone and triciribine could prove to be of therapeutic importance for the clinical treatment of HCC.

## Supporting Information

Figure S1 **Inhibitory effects of 15d-PGJ_2_ on cell proliferation and the stem cell-like phenotype of HCC cells.** A, Huh7, SK-Hep1, SMMC 7721 and Hep3B cells were treated with 0.5 µg/ml 15d-PGJ_2_ for 24 hours. The expression of stemness-related genes was evaluated by quantitative RT-PCR analysis. Data are means ± S.E.M. (n = 3). B, Huh7 cells were treated with 0.5 µg/ml 15d-PGJ_2_ for 48 hours. The percentage of apoptotic cells was evaluated by Annexin V-FITC/7-AAD staining. Data are the means ± S.E.M. (n = 3).
(TIF)

Figure S2 **PPARγ promotes ROS generation to inhibit stem cell-like properties in HCC cells.** A, Huh7 and SK-Hep1 cells were treated with 15d-PGJ_2_ and NAC either alone or in combination for 72 hours, after which they were labeled with DHE and analyzed by flow cytometry. Columns, means (n = 3); bars, S.E.M. *, *P*<0.05; **, *P*<0.01, versus control cells. B, Huh7 cells were cultured in spheroid-forming medium with 15d-PGJ_2_ (1 µg/ml) and NAC (10 mM) either alone or in combination for 7 days, after which spheroid formation was examined. Scale bar = 100 µm. C, Isolated CD133^+^ Huh7 cells and SK-Hep1 cells were treated with 15d-PGJ_2_ (0.5 µg/ml) and NAC (10 mM) either alone or in combination for 24 hours. The expression of stemness-related genes was measured by quantitative RT-PCR. Data shown represent the means ± S.E.M (n = 3).
(TIF)

Figure S3 **PPARγ is partially involved in 15d-PGJ_2_-induced ROS generation.** A, Huh7 cells were transfected with negative control siRNA or PPARγ siRNA-1 or siRNA-2. After 24 hours, total protein was extracted for analysis of PPARγ and GAPDH expression. B, Huh7 cells were transfected with negative control siRNA or PPARγ siRNA-1, after which cells were treated with 0.5 µg/ml 15d-PGJ_2_. Intracellular ROS production was analyzed 72 hours later by flow cytometry (means ± S.E.M, n = 3). *, *P*<0.05; **, *P*<0.01.
(TIF)

Figure S4 **15d-PGJ_2_ induces the generation of ROS through NOX2.** A, Huh7 and SK-Hep1 cells were transiently transfected with negative control siRNA or NOX2 siRNAs (NOX2 siRNA-1 or NOX2 siRNA-3), after which cells were treated with 0.5 µg/ml 15d-PGJ_2_ for another 72 hours. Intracellular ROS production was analyzed by flow cytometry (means ± S.E.M, n = 3). *, *P*<0.05; **, *P*<0.01. B, Huh7 cells were transfected with negative control siRNA or NOX2 siRNA-1, -2 or -3 for 24 hours, after which total protein was extracted for analysis of NOX2 and GAPDH expression. C, Huh7 cells were transfected with negative control siRNA or NOX2 siRNA-1, -2 or -3. The expression of NOX2 mRNA was measured 24 hours later by quantitative RT-PCR. Data are means ± S.E.M. (n = 3).
(TIF)

Table S1 **List of the primers for RT-PCR.**
(PDF)

Table S2 **List of the sequences for siRNA.**
(PDF)
