# Supplementary figures and images for: Inhibition of Oxidative Stress-Elicited AKT Activation Facilitates PPARγ Agonist-Mediated Inhibition of Stem Cell Character and Tumor Growth of Liver Cancer Cells
Source: PLoS One. 2013 Aug 30;8(8):e73038. doi: 10.1371/journal.pone.0073038 (PMC3758331; doi:10.1371/journal.pone.0073038)

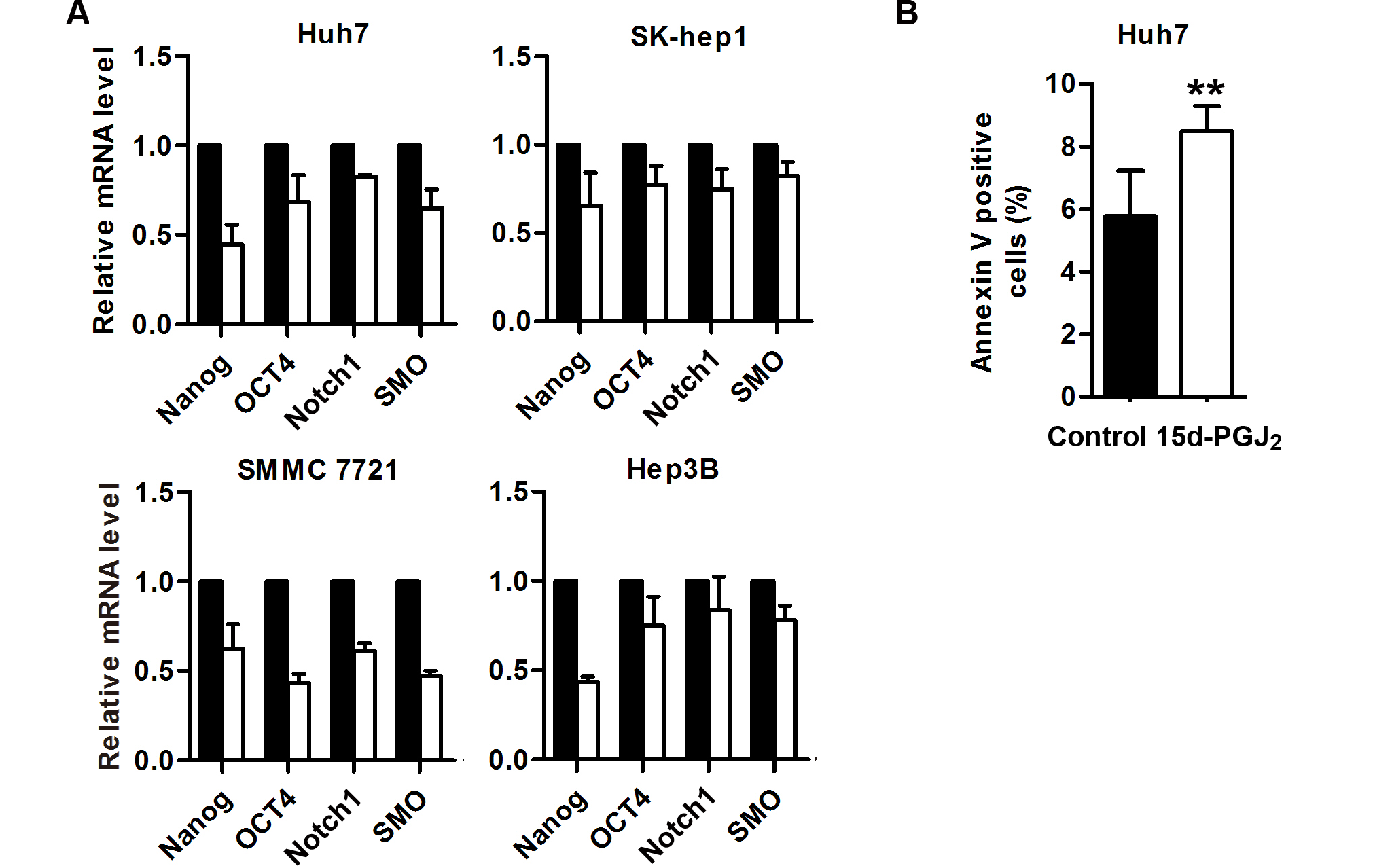

Supplement: Figure S1 — Inhibitory effects of 15d-PGJ2 on cell proliferation and the stem cell-like phenotype of HCC cells. A, Huh7, SK-Hep1, SMMC 7721 and Hep3B cells were treated with 0.5 µg/ml 15d-PGJ2 for 24 hours. The expression of stemness-related genes was evaluated by quantitative RT-PCR analysis. Data are means ± S.E.M. (n = 3). B, Huh7 cells were treated with 0.5 µg/ml 15d-PGJ2 for 48 hours. The percentage of apoptotic cells was evaluated by Annexin V-FITC/7-AAD staining. Data are the means ± S.E.M. (n = 3). (TIF) [file pone.0073038.s001.tif]

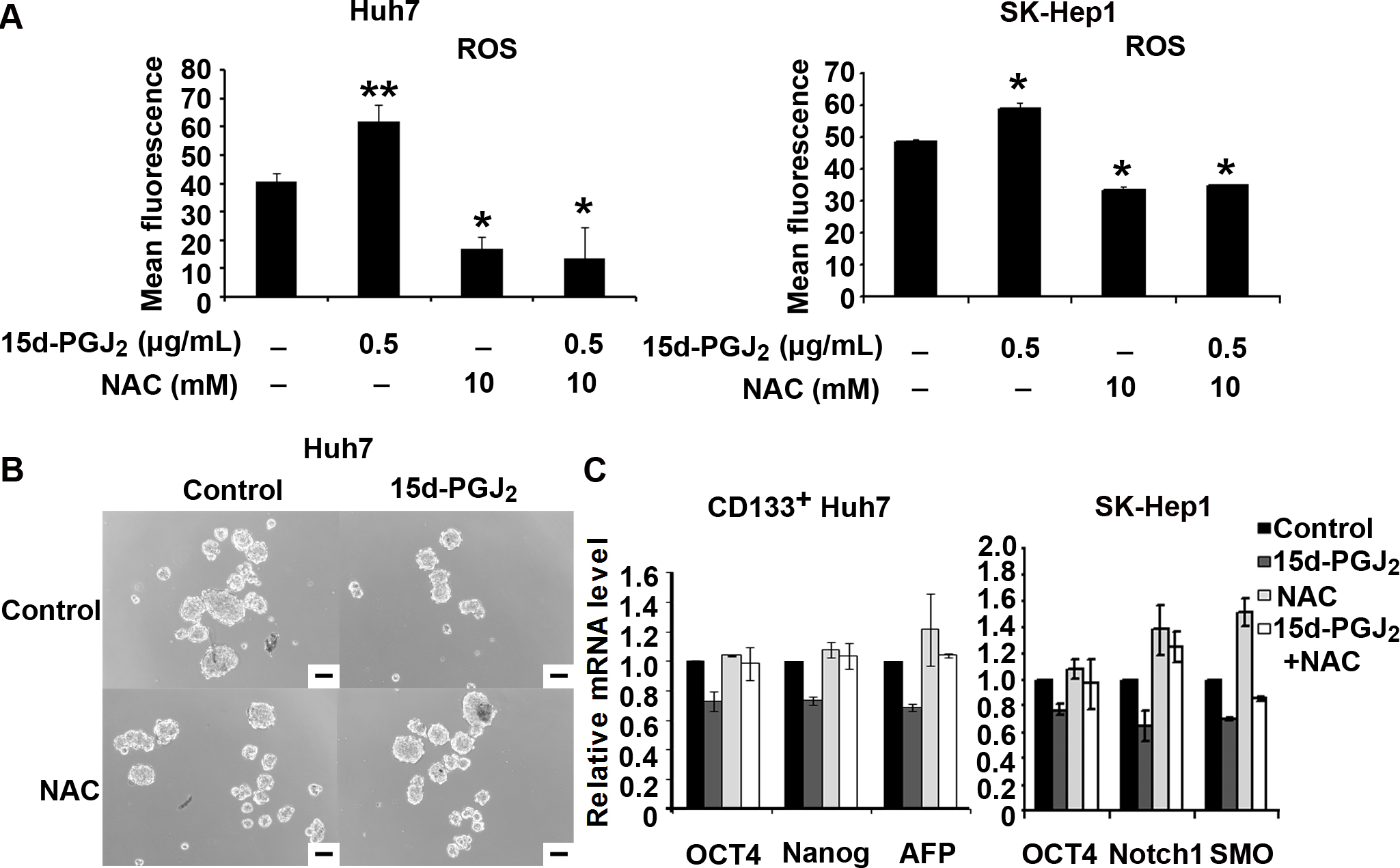

Supplement: Figure S2 — PPARγ promotes ROS generation to inhibit stem cell-like properties in HCC cells. A, Huh7 and SK-Hep1 cells were treated with 15d-PGJ2 and NAC either alone or in combination for 72 hours, after which they were labeled with DHE and analyzed by flow cytometry. Columns, means (n = 3); bars, S.E.M. *, P<0.05; **, P<0.01, versus control cells. B, Huh7 cells were cultured in spheroid-forming medium with 15d-PGJ2 (1 µg/ml) and NAC (10 mM) either alone or in combination for 7 days, after which spheroid formation was examined. Scale bar = 100 µm. C, Isolated CD133+ Huh7 cells and SK-Hep1 cells were treated with 15d-PGJ2 (0.5 µg/ml) and NAC (10 mM) either alone or in combination for 24 hours. The expression of stemness-related genes was measured by quantitative RT-PCR. Data shown represent the means ± S.E.M (n = 3). (TIF) [file pone.0073038.s002.tif]

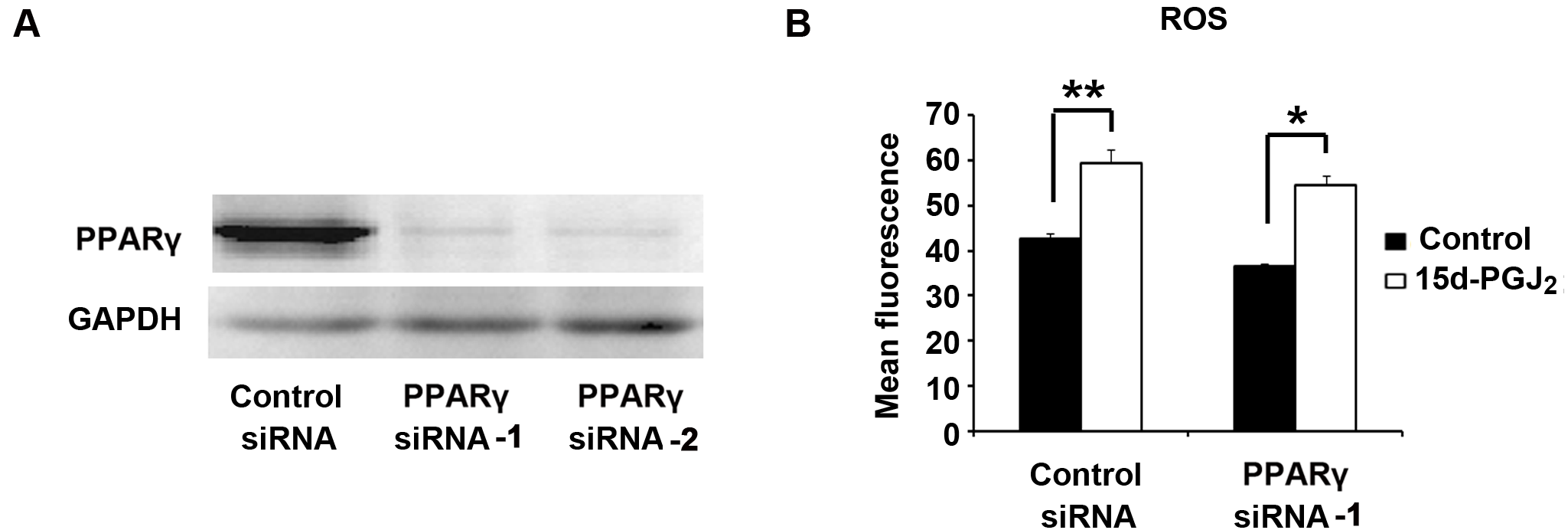

Supplement: Figure S3 — PPARγ is partially involved in 15d-PGJ2-induced ROS generation. A, Huh7 cells were transfected with negative control siRNA or PPARγ siRNA-1 or siRNA-2. After 24 hours, total protein was extracted for analysis of PPARγ and GAPDH expression. B, Huh7 cells were transfected with negative control siRNA or PPARγ siRNA-1, after which cells were treated with 0.5 µg/ml 15d-PGJ2. Intracellular ROS production was analyzed 72 hours later by flow cytometry (means ± S.E.M, n = 3). *, P<0.05; **, P<0.01. (TIF) [file pone.0073038.s003.tif]

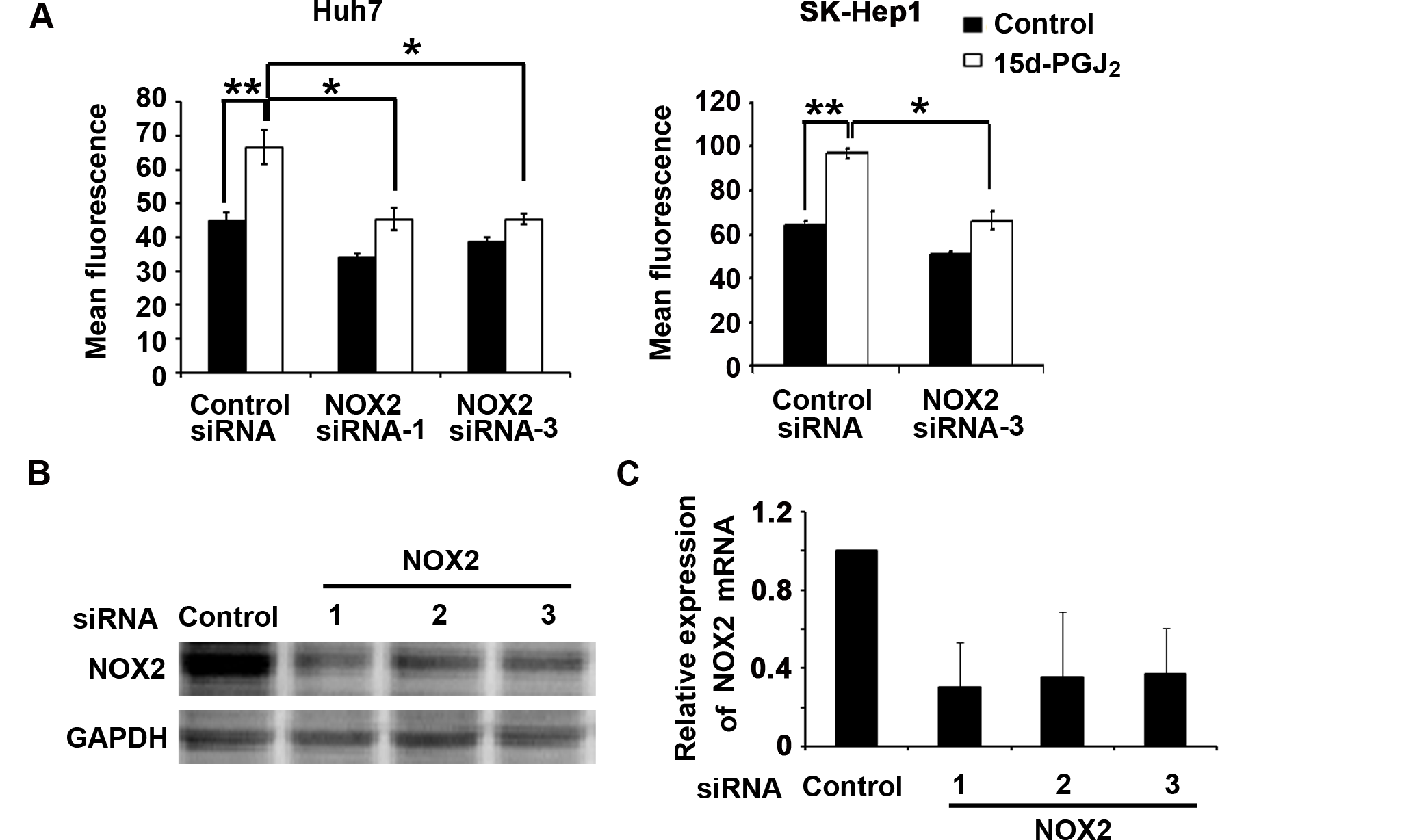

Supplement: Figure S4 — 15d-PGJ2 induces the generation of ROS through NOX2. A, Huh7 and SK-Hep1 cells were transiently transfected with negative control siRNA or NOX2 siRNAs (NOX2 siRNA-1 or NOX2 siRNA-3), after which cells were treated with 0.5 µg/ml 15d-PGJ2 for another 72 hours. Intracellular ROS production was analyzed by flow cytometry (means ± S.E.M, n = 3). *, P<0.05; **, P<0.01. B, Huh7 cells were transfected with negative control siRNA or NOX2 siRNA-1, -2 or -3 for 24 hours, after which total protein was extracted for analysis of NOX2 and GAPDH expression. C, Huh7 cells were transfected with negative control siRNA or NOX2 siRNA-1, -2 or -3. The expression of NOX2 mRNA was measured 24 hours later by quantitative RT-PCR. Data are means ± S.E.M. (n = 3). (TIF) [file pone.0073038.s004.tif]
