# Supplementary material for: Inhibition of Oxidative Stress-Elicited AKT Activation Facilitates PPARγ Agonist-Mediated Inhibition of Stem Cell Character and Tumor Growth of Liver Cancer Cells
Source: PLoS One. 2013 Aug 30;8(8):e73038. doi: 10.1371/journal.pone.0073038 (PMC3758331; doi:10.1371/journal.pone.0073038)
Supplement: Table S1 — List of the primers for RT-PCR. (PDF) [file pone.0073038.s005.pdf]

NAME	Primer (5'-3')	Annealing temperature (℃)	
hNanog-qF<br>hNanog-qR<br>hNanog-Probe	CCAGCTGTGTGTACTCAATGATAG<br>ATTGCTATTCTTCGGCCAGTTG<br>ACCTCAGCCTCCAGCAGATGCAAGA	59	Taqman PCR for human Nanog
hOCT4-qF<br>hOCT4-qR<br>hOCT4-Probe	GTGGTCCGAGTGTGGTTCTGT<br>GCATAGTCGCTGCTTGATCG<br>ACCGGCGCCAGAAGGGCAA	60	Taqman PCR for human OCT4
hAFP-qF<br>hAFP-qR<br>hAFP-Probe	TTACAAAATGCGTTTCTCGTTGC<br>ACTGGAGTCATTTCATGTCTGATAC<br>AATGTCAGCCGCTCCCTCGCCAC	59	Taqman PCR for human AFP
hGAPDH-qF<br>hGAPDH-qR<br>hGAPDH-Probe	GGACCTGACCTGCCGTCTAG<br>TAGCCCAGGATGCCCTTGAG<br>CCTCCGACGCCTGCTTCACCACCT	61	Taqman PCR for human GAPDH
hNotch1-sybr-s<br>hNotch1-sybr-a	GGCCACCTGGGCCGGAGCTTC<br>GCGATCTGGGACTGCATGCTG	65	SYBR PCR for human Notch1
hSMO -sybr-s<br>hSMO-sybr-a	ATCTCCACAGGAGAGACTGGTTCGG<br>AAAGTGGGGCCTTGGGAACATG	64	SYBR PCR for human SMO
hNOX2-sybr-s<br>hNOX2-sybr-a	ACCTCACTGGCTGGGATGAGTCTC<br>ATCCCAGTTGGGCCGTCCATACA	65	SYBR PCR for human NOX2
hNOX4-sybr-s<br>hNOX4-sybr-a	TGCCCATCTGGTGAATGCCCTCA<br>CCCTGTCAGGCCAGGAACAGTTGT	66	SYBR PCR for human NOX4
$hP22^{phox}$-sybr-s<br>$hP22^{phox}$-sybr-a	TCCTGCATCTCCTGCTCTC<br>CACAGCCGCCAGTAGGTAG	59	SYBR PCR for human $P22^{phox}$
$hP47^{phox}$-sybr-s<br>$hP47^{phox}$-sybr-a	TGCCAACTACGAGAAGACCTC<br>ACAGAACCACCAACCGCTCT	60	SYBR PCR for human $P47^{phox}$
$hP67^{phox}$-sybr-s<br>$hP67^{phox}$-sybr-a	CGGACAAGAAGGACTGGAAG<br>ACATGCAGCCAATGTTGAAG	57	SYBR PCR for human $P67^{phox}$
hRac-sybr-s<br>hRac-sybr-a	CAAGCAGAGGCTTGGCGGGG<br>TGGGGCTTGAAGGGTGGGCT	66	SYBR PCR for human Rac
