# Supplementary material for: Inhibition of Oxidative Stress-Elicited AKT Activation Facilitates PPARγ Agonist-Mediated Inhibition of Stem Cell Character and Tumor Growth of Liver Cancer Cells
Source: PLoS One. 2013 Aug 30;8(8):e73038. doi: 10.1371/journal.pone.0073038 (PMC3758331; doi:10.1371/journal.pone.0073038)
Supplement: Table S2 — List of the sequences for siRNA. (PDF) [file pone.0073038.s006.pdf]

NAME	SEQUENCE OF SIRNA
**NOX2 siRNA-1**	CCUAUGACUUGGAAAUGGA
**NOX2 siRNA-2**	CAAUAAUUCUGAUCCUUAU
**NOX2 siRNA-3**	CUAUGACUUGGAAAUGGAU
**PPARγ siRNA-1**	CUCAUAAUGCCAUCAGGUU
**PPARγ siRNA-2**	CAAUCAGAUUGAAGCUUAU
**AKT1 siRNA**	GUGCCAUGAUCUGUAUUUA
**AKT2 siRNA**	CCAUGAAGAUCCUGCGGAA
